# Digital imaging in the immunohistochemical evaluation of the proliferation markers Ki67, MCM2 and Geminin, in early breast cancer, and their putative prognostic value

**DOI:** 10.1186/s12885-015-1531-3

**Published:** 2015-07-25

**Authors:** Shalaka Joshi, Johnathan Watkins, Patrycja Gazinska, John P. Brown, Cheryl E. Gillett, Anita Grigoriadis, Sarah E. Pinder

**Affiliations:** 1 Department of Research Oncology, King’s College London, Faculty of Life Science and Medicine, Division of Cancer Studies, Bermondsey Wing, Guy’s Hospital, London, UK; 2 Breast Cancer Now Unit, King’s College London, Faculty of Life Science and Medicine, Division of Cancer Studies, Bermondsey Wing, Guy’s Hospital, London, UK; 3 King’s Health Partners Cancer Biobank, King’s College London, Faculty of Life Science and Medicine, Division of Cancer Studies, Bermondsey Wing, Guy’s Hospital, London, UK; 4 Present address: Tata Memorial Centre, Dr E Borges Road, Parel, Mumbai, 400012 Maharashtra India

**Keywords:** Ki67, MCM2, Geminin, Proliferation, Digital microscopy, Immunohistochemistry, Survival analysis

## Abstract

**Background:**

Immunohistochemical assessment of proliferation may provide additional prognostic information in early breast cancer. However, due to a lack of methodological standards proliferation markers are still not routinely used for determining therapy. Even for Ki67, one of the most widely-studied markers, disagreements over the optimal cutoff exist. Improvements in digital microscopy may provide new avenues to standardise and make data more reproducible.

**Methods:**

We studied the immunohistochemical expression of three markers of proliferation: Ki67, Mini-Chromosome Maintenance protein 2 and Geminin, by conventional light microscope and digital imaging on triplicate TMAs from 309 consecutive cases of primary breast cancers. Differences between the average and the maximum percentage reactivity in tumour cell nuclei from the three TMA cores were investigated to assess the validity of the approach. Time-dependent Receiver Operating Characteristic curves were utilized to obtain optimal expression level cut-offs, which were then correlated with clinico-pathological features and survival.

**Results:**

High concordance between conventional and digital scores was observed for all 3 markers (Ki67: r_s_ = 0.87, *P* < 0.001; MCM2: r_s_ = 0.94, *P* < 0.001; and Geminin: r_s_ = 0.86, *P* < 0.001; Spearman’s rank). There was no significant difference according to the number of TMA cores included for either Ki67 or MCM2; analysis of two or three cores produced comparable results. Higher levels of all three proliferation markers were significantly associated with higher grade (*P* < 0.001) and ER-negativity (*P* < 0.001). Optimal prognostic cut-offs for percentage expression in the tumour were 8 %, 12 and 2.33 % for Ki67, MCM2 and Geminin respectively. All 3 proliferation marker cutoffs were predictive of 15-year breast cancer-specific survival in univariable Cox regression analyses. In multivariable analysis only lymph node status (HR = 3.9, 95 % CI = 1.79-8.5, *P* = 0.0006) and histological grade (HR = 1.84, 95 % CI = 1–3.38, *P* = 0.05) remained significantly prognostic.

**Conclusions:**

Here we show that. MCM2 is a more sensitive marker of proliferation than Ki67 and should be examined in future studies, especially in the lymph node-negative, hormone receptor-positive subgroup. Further, digital microscopy can be used effectively as a high-throughput method to evaluate immunohistochemical expression.

**Electronic supplementary material:**

The online version of this article (doi:10.1186/s12885-015-1531-3) contains supplementary material, which is available to authorized users.

## Background

Breast cancer is a heterogeneous disease [1]. With earlier detection and improved treatment options, breast cancer-related mortality is decreasing, while the detection of early stage disease is on the rise [2]. Traditional prognostic and predictive factors such as lymph node status, histological grade, invasive tumour size, hormone receptor (ER and PR) and HER2 status may be insufficient for prognosticating early stage disease [3, 4]. As such, there is a need for better markers to categorise primary, operable breast cancers and reduce overtreatment in those patients with a good prognosis, and offer more aggressive treatment regimes to those in the poor prognosis group.

Proliferation is one of the most fundamental properties of cancer [5]. Histological grade is an important prognostic marker, which reflects proliferation status by incorporating an assessment of mitotic rate. Other methods of assessing proliferation, such as S-phase fraction, mitotic activity index (MAI) and radionucleotide labeling indices have limitations, and have not proven to be of utility over and above the prognostic value of histological grade, and consequently, they have not been applied in clinical practice [6]. Ki67 has been one of the most extensively studied proliferation markers since its discovery in the early 1980s [7]. Since the development of the MIB-1 antibody, immunohistochemical expression of Ki67 in paraffin-embedded tissue has been shown in a number of studies to be prognostic and predictive of treatment response in breast cancer [8–10].

Molecular profiling of breast cancer can be used to classify early breast cancer into prognostic groups [1]. Ki67 measured by immunohistochemistry (IHC) has been proposed to be a useful surrogate for molecular subtype. Ki67 at a cut-off of 13.25 % can identify and divide ER-positive breast cancers into the luminal A and B subgroups with moderate accuracy and with a significant difference in patient survival [11]. As a result, the St Gallen guidelines recommend a cut-off of 14 % for Ki67 in deciding how to manage early breast cancer patients in the adjuvant treatment setting [12]. Other studies have reported that immunohistochemical analysis of ER, PR, HER-2 and Ki67 (the latter at a cut-off of 10 %), and a derived IHC-4 score is equivalent to the 21-gene recurrence score that is the basis of Oncotype-DX in predicting recurrence and survival in ER-positive breast cancer [13, 14]. Currently, trials are underway to stratify hormone receptor-positive, early breast cancer patients by their gene expression profile into those with a low or high risk of recurrence [15], which in turn influences the decision to administer chemotherapy. Of note, 5 of the 21 genes assessed in Oncotype-DX are proliferation genes, emphasising the importance of proliferation status in tumour prognostication and in clinical decision making [16]. Gwin *et al.* studied the correlation of Ki67 expression assessed by IHC in 32 breast cancer patients for possible association with the Oncotype-DX’s recurrence score (RS) and found it to be high in some of the low RS cases, as a result of which they suggested that Ki67 be used alongside the RS [17].

Other markers of proliferation have been identified as participants in the process of DNA replication as well as exhibiting prognostic value. Mini-chromosome maintenance (MCM) proteins are DNA helicases that, along with the Origin Recognition Complex (ORC) and Cdc6p, form the pre-Replication Complex (pre-RC), to initiate DNA replication [18]. The dissociation of MCM proteins from the pre-RC is controlled by Geminin, which prevents re-replication by inhibiting Cdt-1 [19]. The immunohistochemical expression of these proteins has been correlated with prognosis in breast and other cancers [20–22].

However, methodological variability in assessing these proliferation markers represents one of the main difficulties for translating these research findings into the clinic. Consequently, in an attempt to standardise the technique, the “International Ki67 in Breast Cancer Working Group” has drafted guidelines for the immunohistochemical assessment of Ki67 [23]. Adhering to these criteria, we carried out a study to evaluate two different methods of assessing Ki67, MCM2 and Geminin IHC in tissue microarrays (TMAs) of a series of consecutive invasive breast cancer cases. We aimed to evaluate the concordance between conventional microscopic methods (i.e. the histological sections) and digital scanned images from the same material applied to three markers of proliferation. Having evaluated the similarity between the two scoring methodologies, we sought to compare the expression patterns of the three proliferation markers with each other in order to establish their ability to capture tumour proliferation status, as well as to determine their association with clinico-pathological characteristics.

## Methods

### Patients

Formalin-fixed paraffin-embedded (FFPE) tissue blocks were retrieved from 309 patients who presented with primary invasive breast cancer between December 1989 and September 1992 to Guy’s and St Thomas’ Breast Unit. Unless there was insufficient tissue for research purposes, consecutive cases were selected, All patients were treated surgically, either in the form of modified radical mastectomy or breast conservation surgery, followed by adjuvant treatment. Written, informed consent was obtained before procuring the tissue for research purposes. Permission to use samples and data was given by the Cancer Biobank Access Committee (License number 12121) in accordance with NHS Research Ethics Committee conditions.

### Tissue Microarrays (TMAs) and Immunohistochemistry (IHC)

Tissue samples were uniformly fixed in 10 % formalin within 30 min of surgery. Representative areas were marked on H & E sections for TMA construction. TMAs were made in triplicate using a manual arrayer (Beecher Instruments, Sun Prairie, WI, USA) with 0.6 mm stylet. Each TMA consisted of 85–115 tissue cores, with 5 cores of control tissue samples placed strategically within the block to enable orientation. Sections were cut at 3 μm and floated onto polyanionic slides before being dried at 37 °C overnight followed by incubation for 2 h at 56 °C. The TMA sections were obtained during the study and freshly stained, as per the recommendations. They were then incubated with the antibodies after establishing appropriate IHC protocols. A two-step, compact, polymer chain, biotin-free IHC protocol on the BOND-MAX^TM^ (Leica Biosystems, UK) staining system was used with a primary antibody incubation time of 30 min. Antigen retrieval was performed using BOND-MAX Epitope Retrieval solution 1 (Leica Biosystems, UK). The chromogen used was 3,3^′^-diaminobenzidine (DAB). ER and HER2 status were obtained from patient records. The antibodies are listed in Table 1.

**Table 1:** Antibody panel used for immunohistochemistry

Antigen	Clone	Dilution	Source	System	Scoring method
Ki67	MIB1	1 in 75	Leica	Leica, BOND-Max	As described
MCM2	CRCT2.1	1 in 100	Leica	Leica, BOND-Max	As described
Geminin	EM6	1 in 30	Leica	Leica, BOND-Max	As described
ER	SP6	1 in 100	Invitrogen	Leica, BOND-Max	>2 Allred
HER2	Ready to use kit		Leica	Leica, BOND-Max	3+

### Scoring the immunohistochemical expression of proliferation markers: conventional and digital imaging

For each of the three markers, a score was determined by assessment of the percentage of invasive carcinoma cells with positively staining nuclei. At least 50 tumour cells per TMA core were considered necessary to ascertain a representative score. Any cores that were folded, absent, or contained an inadequate number of tumour cells were not scored. Conventional scoring was conducted with an Olympus BX50 microscope (Olympus Optical Co., Ltd., Tokyo, Japan) by the first author (SJ) after a period of training and joint scoring.

Slides were subsequently scanned using a Nanozoomer (Hammamatsu, UK), transferred to the digital slide server and accessed online via the Slidepath system (Leica Biosystems, UK). Digital microscopic scoring was performed with the OpTMA scoring software platform (Leica Biosystems, UK) and the percentage of positive nuclei was again assessed similarly to the light microscopic slides. Scoring using each of the two methods was performed independently by the same reader (SJ), one method at a time, and blinded to the results of assessment by the other method. Approximately 10 % of the scores were assessed by more than one author (SJ, JB, PG) and there was in general good agreement among the authors. Since the TMAs were assessed in triplicate, both the maximum (from the 3 cores) and the average of the 3 scores were recorded for final analysis.

### Statistical methods

Where tumours were categorised into two continuous groups, the significance of associations of each of the immunohistochemical scores was assessed with a Mann Whitney test. For clinico-pathological features that grouped tumours into three or more continuous, unpaired categories, a Kruskal-Wallis test was used to assess association. To analyse associations between two continuous variables, Spearman’s rank correlation was applied. Wilcoxon signed rank test and Friedman’s test were used to evaluate continuous, paired variables of 2 and 3 groups, respectively. All the above statistics were performed using GraphPad PRISM Version 6.0c (GraphPad Software, Inc, CA, USA).

In order to establish a cut-off between high and low expression that enabled the most accurate prediction of breast cancer-specific survival (BCSS) for each of the markers, time-dependent Receiver Operating Characteristic (ROC) curves were created from the censored survival data using the Kaplan-Meier method with the R package survivalRO [24]. The sensitivity and specificity for predicting 15-year BCSS were calculated for various cut-off values using a statistically-determined baseline marker value as reference [25]. The value that yielded the highest balanced accuracy, defined as (sensitivity + specificity)/2, was selected as the optimal cut-off value.

Using the defined cut-off values to categorise cases into high-expressing and low-expressing tumours, Kaplan-Meier survival curves were constructed and compared using the log-rank test for each marker. BCSS was defined as the interval from the date of histological diagnosis to the date of death due to breast cancer up until 15 years. All other causes of death, including those cases where the cause was unknown or was ambiguous, were censored at the last follow-up.

Multivariable analysis was conducted using Cox’s regression model with backward stepwise model selection of predictors using the Akaike Information Criterion [26]. The initial set of predictors for the multivariable model included histological grade (1, 2 or 3), age (>50 years or <50 years), lymph node status (positive or negative), clinical tumour size (<2 cm, 2–5 cm or >5 cm), ER status (Allred > 2 as positive) and HER2 status (positive if scored 3+ on IHC or FISH positive). Multivariable analysis was then conducted as before. Subgroup univariable and multivariable survival analyses on ER-positive cases were conducted similarly. All survival analysis was performed in the statistical language R and is provided as a Sweave document in Supplementary Methods (Additional file 3). In all statistical tests, *P* < 0.05 was considered significant.

## Results

### Patient and tumour characteristics

Patient and tumour characteristics are shown in Table 2. In this series of 309 cases, 70.1 % of patients were over 50 years of age, 53.8 % had lymph node-negative disease, 75.6 % were ER-positive and 16.8 % were HER2-positive (although HER2 status was known for only 50 % of patients in this historical cohort). 43.4 % were of histological grade 2 and 55.4 % were between 2 and 5 cm in size. The median follow-up period was 13 years (1 to 17.2 years). The median overall survival was 13.48 years (0.3 to 18.1 years). There were 160 patients who died (51.8 %) at the end of the follow up period, only 83 of whom were known to have died of breast cancer.

**Table 2:** Patient and tumour characteristics of 309 cases of early breast cancer

Clinico-pathological feature	Distribution (percentage of cases with data)
Age, years	
Median	58
Range	28−85
<50	92 (29.9 %)
>50	216 (70.1 %)
Tumour size	
<2 cm	114 (41.3 %)
2−5 cm	153 (55.4 %)
>5 cm	9 (3.3 %)
Not known	33
LN status	
Positive	132 (46.2 %)
Negative	154 (53.8 %)
Not known	23
Histological Grade	
1	56 (20.1 %)
2	121 (43.4 %)
3	102 (36.6 %)
Not known	30
ER (Estrogen Receptor) status	
Positive	226 (75.6 %)
Negative	73 (24.4 %)
Not known	10
HER2 status (IHC 3+ or FISH + ve)	
Positive	26 (16.8 %)
Negative	129 (83.2 %)
Not known	154
Recurrence (Local, regional, distant or death when death was known to be caused by breast cancer)	
Total	111/309 (35.9 %)
Median time to recurrence (years)	3.14
Range (years)	0.05−19.05
Mortality	
Total deaths with known cause	148
Deaths due to breast cancer	83 (56 %)
Deaths with breast cancer present at death	57 (38.5 %)
Deaths due to causes other than breast cancer	8 (5.4 %)
Not known	12
Overall survival (years)	
Median	13.48
Range	0.33−18.11
Follow-up (years)	
Median	13
Range	1−17.2

### Correlation between proliferation markers and methodology

**Fig. 1:**
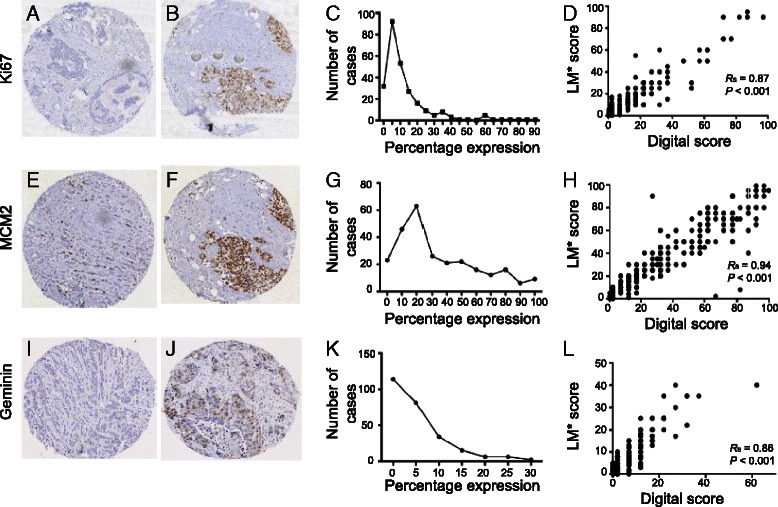
Expression of proliferation markers in invasive breast cancers. Representative breast cancer cores from a consecutive TMAs showing low and high immunohistochemical staining for 3 proliferation markers Ki67 (**a,b**), MCM2 (**e,f**) and Geminin (**i,j**) (150X magnification). Distribution of IHC determined expression of Ki67 (**c**), MCM2 (**g**) and Geminin (**k**) across 309 primary breast carcinomas. The number of cases is indicated on the x-axis, while the percentage scoring for the respective marker is depicted in the y-axis. Correlation between light microscopic and digital image guided scores for Ki67 (**d**), MCM2 (**h**) and Geminin (**l**). The Spearman’s rank correlation coefficient and *p*-values are shown

To explore the information provided by the scores for each marker, we first compared them across the cohort. We found that a greater proportion of tumour cells showed expression of MCM2 than Ki67 and Geminin, with the latter having the lowest frequency of expression (*P* < 0.001; Wilcoxon signed rank test). The median light microscopic scores of Ki67, MCM2 and Geminin when using the maximum score from the 3 TMA cores, were 10 %, 30 and 5 %, respectively. With the mean light microscopic score from the 3 cores, the median values of Ki67, MCM2 and Geminin expression were 7.7 %, 24 and 3 %, respectively. With the digital scoring technique, the medians of the maximum scores from the 3 TMA cores were 7 %, 37 and 2 % whereas the medians of the average 3 scores were 4.5 %, 27 and 2 % for Ki67, MCM2 and Geminin, respectively (Table 3). Representative cores with staining for Ki67, MCM2 and Geminin are shown in Fig. 1a-b, e-f and i-j, respectively. Frequency distribution curves for the average Ki67, MCM2 and Geminin scores are shown in Fig. 1c, g and k, respectively.

**Table 3:** Immunohistochemical expression of Ki67, MCM2 and Geminin in 309 cases of early breast cancer as assessed by light microscope and digital imaging and the correlation between the two methods of scoring

Marker	Score	Conventional method of scoring				Digital method of scoring				Correlation
		Available values	Max	Min	Median	Available values	Max	Min	Median	Spearman’s co-efficient
Ki67	Maximum of the 3 cores	258	95	0	10	175	97	0	7	*n* = 174^a^
										0.90 (0.86−0.92)
										*p* < 0.001
	Average of the 3 cores	258	90	0	7.7	175	85.33	0	4.5	*n* = 174^a^
										0.91 (0.88−0.93)
										*p* < 0.001
MCM2	Maximum of the 3 cores	260	100	0	30	167	100	0	37	*n* = 167^a^
										0.92 (0.90−0.94)
										*p* < 0.001
	Average of the 3 cores	260	100	0	24	167	98.5	0	27	*n* = 167^a^
										0.94 (0.91−0.95)
										*p* < 0.001
Geminin	Maximum of the 3 cores	258	40	0	5	270	62	0	2	*n* = 257
										0.88 (0.85−0.91)
										*p* < 0.001
	Average of the 3 cores	258	28.3	0	3	270	37	0	2	*n* = 257
										0.90 (0.87−0.92)
										*p* < 0.001

^a^The number of cores available for digital scoring was not the same as the number available for scoring conventionally. Hence, only those scored by both techniques were compared with each other

In order to assess inter-core variability within a sample, we compared the expression of Ki67 (110 cases), MCM2 (116 cases) and Geminin (105 cases) across those samples for which all 3 cores were available and found no significant difference for Ki67 or MCM2, (*P* = 0.411 for Ki67, *P* = 0.322 for MCM2; Friedman’s test) indicating that Ki67 and MCM2 expression was consistent across the 3 cores. In contrast, the inter-core variability for Geminin was significantly higher (*P* < 0.006; Friedman’s test). Of note, the average of 2 cores provided comparable results to the average values of 3 cores (Ki67: r_s_ = 0.96, *P* < 0.0001; MCM2: r_s_ = 0.95, *P* < 0.0001; Geminin: r_s_ = 0.95, *P* < 0.0001) suggesting that one may evaluate 2 or 3 cores for such IHC markers. We also observed that the loss of data due to core loss or absence of sufficient tumour, decreased from 37−40 % to 22 and 16 %, if 1, 2 or 3 cores were considered respectively for all 3 proliferation markers. The average of the values obtained from 3 cores strongly correlated with the maximum of the 3 (Ki67: r_s_ = 0.97, *P* < 0001; MCM2: r_s_ = 0.98, *P* < 0.0001; Geminin: r_s_ = 0.98, *P* < 0.0001). Since there was little difference between the average and maximum value obtained from 3 cores; we proceeded with the average value for further analysis.

### Comparison between conventional light microscopic and digital image assessment

We next asked whether there was any appreciable difference between the results obtained from scoring the section using the traditional light microscope as opposed to assessment of the scanned digital image. A significant correlation between the scores of the two techniques was observed for each marker (Ki67: r_s_ = 0.87, *P* < 0.001, Fig. 1d; MCM2: r_s_ = 0.94, *P* < 0.001 Fig. 1h; and Geminin: r_s_ = 0.86, *P* < 0.001, Fig. 1l; Spearman’s rank correlation), with the scores for MCM2 exhibiting the highest concordance.

### Association with clinico-pathological features and BCSS

We investigated whether the immunohistochemical expression of Ki67, MCM2 and Geminin was significantly associated with clinico-pathological features. These analyses were performed using the median value of both the maximum as well as the average values of three TMA cores scores and no significant difference between these two approaches was observed. Whilst tumour size, lymph node status and HER2 status were not associated with any of the three proliferation markers, higher histological grade and ER-negative tumours had higher expressions of all 3 markers, *P* < 0.001 for all, Mann Whitney test (Table 4).

**Table 4:** Association between the proliferation markers Ki67, MCM2 and Geminin and other prognostic factors in 309 cases of early breast cancer

Clinico-pathological Feature	Categories	Ki67 median	*p*-value	MCM2 median	*p*-value	Geminin Median	*p*- value
Age^a^	</= 50 years	10	0.006	31.6	0.097	3.4	0.101
	>50 years	7		21.3		3	
Grade^b^	1	4.5	<0.001	16.5	<0.001	0.5	<0.001
	2	7		20.7		2.7	
	3	14.7		50		6.3	
ER status^a^	Positive	6.7	<0.001	19.1	<0.001	2.3	<0.001
	Negative	14.6		50		8.1	
LN status^a^	Positive	7.5	0.787	24	0.734	3	0.387
	Negative	8		24.67		3	
HER2^ac^ status	Positive	9	0.299	30	0.298	3	0.039
	Negative	12.7		37		8.3	
Tumour size^b^	</= 2 cm	7.5	0.034	23.4	0.197	2.6	0.132
	>2, < 5 cm	7.1		24.3		3	
	>/= 5 cm	14.5		37.5		5.7	

^a^Mann Whitney test used to test the association between 2 continuous, unpaired variables

^b^Kruskal-Wallis test used to test the association among 3 continuous, unpaired variables

^c^Only 155 cases with known HER2 status were included to test the association of HER2 status with each of the proliferation markers

**Fig. 2:**
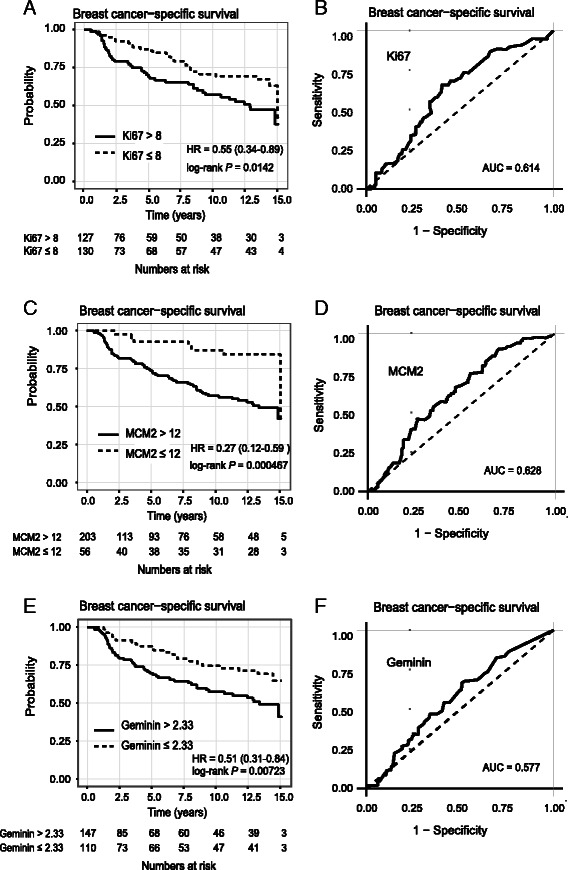
Univariable breast cancer-specific analyses among 309 invasive breast carcinomas. Kaplan Meier curves showing breast cancer-specific survival (BCSS) in relation to high (solid line) and low (dotted line) expression of Ki67 (**a**), MCM2 (**c**) and Geminin (**e**). The cut-offs of percentage expression were 8, 12 and 2.33 for Ki67, MCM2 and Geminin, respectively. Log rank *p*-values are stated. The number of patients at risk for every 2.5 years is given for each subgroup. Using time-dependent Receiver Operating Characteristic (ROC) curves for 15-year BCSS, optimal cut-offs were calculated for Ki67 (**b**), MCM2 (**d**) and Geminin (**f**)

Next we investigated if any of the three markers of proliferation possessed prognostic value in our cohort by first using time-dependent ROC curves to calculate cut-offs that yielded the highest balanced accuracy for 15-year BCSS. These cut-offs were 8 %, 12 and 2.33 % for Ki67, MCM2 and Geminin, respectively (ROC curves for cut-off calculation are shown in Fig. 2b, d and f). In a univariable Cox regression analysis, high expression of all 3 markers of proliferation was significantly associated with 15 year BCSS using optimal cut-off values for Ki67 {*P* = 0.0142, HR = 0.55 (0.34−0.89); log-rank test showing 95 % confidence intervals} (Fig. 2a); for MCM2 {*P* = 0.0005, HR = 0.27 (0.12−0.59); log-rank test showing 95 % confidence intervals} (Fig. 2c); and for Geminin {*P* = 0.0072, HR = 0.51 (0.31−0.84); log-rank test showing 95 % confidence intervals} (Fig. 2e).

To offset some of the heterogeneity that arises from the inclusion of ER/PR negative cases in a consecutive series of patients, we next used the same expression cut-offs and looked within the ER-positive subgroup. We recapitulated the results seen in the wider cohort with Ki67 {*P* = 0.049, HR = 0.53 (0.28−1.01); log-rank test showing 95 % confidence intervals} (Additional file 1A) having the weakest prognostic value, MCM2 the strongest {*P* = 0.0148, HR = 0.35 (0.15−0.85); log-rank test showing 95 % confidence intervals} (Additional file 1B), followed by Geminin {*P* = 0.0254, HR = 0.47 (0.24−0.93); log-rank test showing 95 % confidence intervals} (Additional file 1C).

To examine the utility of these markers as independent predictors of survival, we also performed multivariable Cox regression analysis with backward stepwise regression, and found only high histological grade {*P* = 0.0502, HR = 1.84 (1–3.38)} and lymph node-positive status {*P* = 0.0006, HR = 3.9 (1.79−8.5)} to be associated with breast cancer-related death within 15 years for all breast cancers irrespective of ER positivity (Table 5). Among ER-positive cases, again only lymph node-positive status {*P* = 0.0006, HR = 7.13 (2.32−21.89)} remained significantly associated with BCSS following a multivariable analysis (Additional file 2).

**Table 5:** Univariable and multivariable analyses of prognostic factors for 15-year breast cancer specific survival in 309 cases of early, invasive breast cancer

Prognostic factor	Univariable Cox regression analysis			Multivariable Cox regression analysis		
	HR	95 % CI	*p*-value	HR	95 % CI	*p*-value
Ki67: low	0.55	0.34−0.89	0.014	1.38	0.56−3.38	0.485
MCM2: low	0.27	0.12−0.59	0.0004	0.352	0.07−1.85	0.218
Geminin: low	0.51	0.31−0.84	0.007	0.824	0.34−2.02	0.673
Histological grade: 3	1.95	1.41−2.69	<0.0001	1.42	0.68−2.96	0.346
ER status: positive	0.32	0.2−0.5	<0.0001	0.737	0.32−1.72	0.48
HER2 status: positive	1.65	0.84−3.25	0.143	0.545	0.2−1.5	0.241
LN status: positive	3.35	1.98−5.66	<0.0001	3.44	1.54−7.72	0.002
Age > 50 year	0.85	0.53−1.34	0.477	1.36	0.63−2.91	0.431
Tumour size: medium	0.76	0.23−2.49	0.891	1.38	0.16−12	0.772
Tumour size: small	0.8	0.25−2.6	0.891	2.84	0.32−25.35	0.35
After backward stepwise regression						
Grade: 3	1.84	1−3.38	0.0502			
LN status: positive	3.9	1.79−8.5	0.0006			

## Discussion

We have assessed TMAs of 309 cases of primary invasive breast cancers for the expression of the proliferation markers Ki67, MCM2 and Geminin by IHC using conventional light microscopy and by digital imaging. We observed a significantly positive correlation between the methodologies in assessing all the 3 biomarkers confirming that remote assessment of scanned images is comparable with using light microscopy to score histological glass slides.

The methodological aspects of immunohistochemistry are being increasingly standardised as a consequence of the widespread uptake of automated systems that improve consistency. By extending this approach to include digital imaging and computer-aided systems it may be possible to confer greater objectivity to methods of immunohistochemical scoring [27]. In agreement with our findings, and with a view to implementing these changes, Konsti *et al*. have developed a virtual microscopy and automated analysis platform, which showed 87 % agreement and a weighted kappa value of 0.57 when compared to visual assessment of Ki67 immunohistochemical expression in breast cancer [28]. Digital microscopy for scoring of scanned images of the TMAs, a high-throughput method, has advantages over the conventional light microscopic method. These include ease of handling compared to manual navigation of a TMA slide: for example, the linking of cores to the predefined TMA ‘map’ ensures that the core/case are accurately identified and recorded. In addition, the samples can be accessed and evaluated remotely through any computer without the need for availability of a light microscope and thus this method provides an opportunity to exchange information between observers, such as the double-reading of slides (particularly valuable for clinical trial material), with ease. Voros *et al*. used a partially digitised counting method for Ki67, and concluded that such a technique was faster, more convenient and would significantly improve the reproducibility of using Ki67 as a proliferation marker in breast cancer [29].

In this study, we do not report digital image analysis of the cases using computer software but describe the scoring of proliferation marker-stained scanned images by human observers. One of the goals of automated image analysis would be to improve the accuracy and reproducibility in scoring biomarkers such as Ki67, MCM2 and Geminin. Fasanella *et al*. used computer-assisted image analysis of digitised slides, and found manual and automated methods to be comparable in assessing Ki67 expression in breast cancer [8, 30]. However, in our opinion, further work is required before automated image analysis can be widely adopted for the determination of proliferation marker frequency in invasive breast cancer patients although our results hint at the potential advantages and non-inferiority to the assessment of digital images over conventional means.

We encountered some recurring questions on the approach to, and methodology of, immunohistochemistry in the TMA setting. TMA technology has been widely used in research and some guidelines for practice are now available [31]. Nonetheless, there are some unresolved issues including the optimum number of cores to be assessed, the extraction of a per-sample score from values obtained from multiple cores (maximum or average), and the calculation of an optimal cut-off for prognostication. For Ki67, we found the average score from two cores to be highly correlated with the average score from three cores. For this marker, using either the average or the maximum from the three cores as the final score, we found little difference in their association to clinico-pathological features, implying that either would be appropriate. Moreover, we observed no significant inter-core variability in Ki67 and MCM2 expression, although Geminin expression differed significantly among the 3 cores. We conclude that for each biomarker study, similar analyses are required to evaluate the number of cores required for assessment of that specific lesion, and indeed whether that specific marker can be reliably determined from TMAs at all. Biomarkers with low level expression (such as Geminin) may not be appropriate for TMA studies since reproducible scores from small samples are more problematic than for markers expressed at consistently higher levels (such as MCM2). As a general principle, multiple cores need to be assessed in an attempt to simulate the whole slide and all the representative areas. IHC scoring of a single 1 mm TMA core for ER/PR/HER2 was found to be sufficient, without significant heterogeneity by Kyndi and colleagues [32]. Similarly, estimation of Ki67 using TMAs has been proven to have good concordance with whole section assessment [33]. In practice, most studies, including ours, indicate that triplicate core assessment using a 0.6 mm core size is sufficient for the accurate evaluation of Ki67 and also MCM2 in invasive breast cancer tissue [33, 34].

Different methods of calculating cut-off values for survival analysis have been attempted in the literature, including the dataset median or mean, a literature-informed value, or an even more arbitrary value [8]. The clinical utility of proliferation marker immunohistochemistry has been largely hampered by the lack of consensus with respect to the cut-off used. In a review by Luporsi and colleagues, Ki67 cut points were distributed between 5 and 38 %, with most studies using a cut-off between 10 and 20 % [10]. A multivariable analysis by Tashima *et al*. to determine the optimal cut-off for Ki67 revealed 20 % to be the optimal value [35]. In this study, we used time-dependent ROC curves to find the cut-off that yielded the highest balanced accuracy for 15-year BCSS in this patient cohort [25]. The cut-offs we found were lower than those reported in much of the literature. This may reflect our own patient cohort. In addition, the optimal values we report are those we have found to be associated with BCSS as opposed to overall survival (OS) or disease-free survival (DFS), both of which are vulnerable to confounding factors and which are the outcomes reported in other series [36].

As expected, we found ER-negative and high grade tumours to have significantly higher proliferation indices for all 3 markers [37]. Ki67 expression was also significantly associated with tumour size and patient age although none of the three proliferation markers were associated with lymph node or HER2 status (for the number of cases for whom HER2 status was available). These findings are consistent with those from most studies of proliferation markers in breast and other cancers [9].

**Fig. 3:**
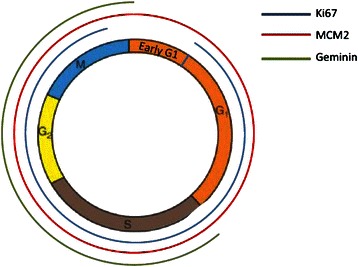
Differential expressions of the three proliferation markers during the cell cycle. Ki67’s expression (shown with a blue line) is detectable from late G1 to M phase. MCM2 (red line) is present in all cell cycle phases. Geminin (green line) is expressed only in the G2-M phase making it a more specific but less sensitive marker of proliferation

The proliferation status of a tumour gives an estimate of the rate at which tumour cells enter the cell cycle, which reflects the rate of tumour growth. Ki67 is expressed from late G1 to M phase, MCM2 in all phases and Geminin in the S-G2-M phases of the cell cycle (Fig. 3). This theoretically makes MCM2 a much more *sensitive* marker of proliferation, since it detects cells that are “licensed to proliferate” and capable of initiating DNA replication [18]. In contrast, Geminin is a more *specific* marker of proliferation, as it only detects cells that are “committed to proliferate” [38]. MCM2 has a significantly higher frequency of expression in breast cancer nuclei than Ki67 and Geminin. Of note, we found MCM2 to be a more robust and sensitive prognostic marker than Ki67 and Geminin in a univariable survival model, which could be a consequence of these markers being differentially expressed during the cell cycle. In agreement with our findings, Gonzalez *et al*. found the MCM2 labelling index to be significantly associated with overall survival and disease free survival in breast cancer and, indeed, that MCM2 was independent of, and superior to, histological grade, Ki67 labelling index and lymph node stage in determining prognosis in a multivariable analysis [20]. Similarly, in a study of oral cavity squamous cell carcinoma, Szelachowska *et al*. found MCM2 to be prognostically superior to Ki67 in predicting 5-year OS [39] whereas the findings of Rodins *et al.* demonstrated MCM2 to be a better marker of proliferation than Ki67 in normal renal epithelial cells and in different types of renal tumours, with Ki67 significantly underestimating the number of dividing cells [40]. A number of studies have shown MCM2 expression to be a significant prognostic marker in other tumour types including oesophageal [41] and laryngeal squamous cell carcinoma [42] and oligodendroglioma of the brain [43]. One possible explanation for these observations is the low expression of Ki67 in early G1 phase, which leads to the fraction of cells at this stage of the cell cycle being missed [40]. It thus remains unclear why Ki67 is so utilised in prognostication in invasive breast cancer and other tumours whilst MCM2 is not routinely used.

One potential shortcoming of our study was that all operable, invasive breast cancer cases were included. Subgroup analyses where the assessment of proliferation may be most clinically relevant, for example, of tumours that were lymph node-negative and hormone receptor-positive were not attempted since there were fewer than 100 cases available in our series. One established cut-off for Ki67, as defined by St Gallen’s guidelines, is 14 % but this is derived from data on hormone receptor-positive patients. We applied this cut-off in our entire dataset and found the two groups of high versus low expressers had significantly different survivals (data not shown) but our series included both receptor negative and positive disease. In this setting therefore we sought to identify an optimum cut-off for a consecutive cohort of all these operable invasive breast cancers.

Although MCM2 appeared to be more strongly associated with BCSS in a univariable analysis than Ki67, none of the three proliferation biomarkers were independent predictors of survival in a multivariable analysis of the whole cohort or of the ER positive sub-population. Lymph node positivity in this series was the most important prognosticator. The greatest utility of assessing proliferation markers may be limited to good prognosis, receptor-positive sub-populations of patients and the clinical utility to select those who are likely to benefit from adjuvant chemotherapy. Of note, different cut-offs may need to be applied to differing sub-types of invasive breast carcinomas to maximise the clinical benefit of determination of proliferation marker expression, which is unlikely to be a ‘one size fits all’. However, there were insufficient patients in this good prognosis sub-group for such an approach to be confirmed in the present study.

Inter-observer variation is another critical issue with immunohistochemistry although most studies have shown a good concordance rate. We did not assess inter-observer variation in our study, although there was a general agreement regarding the score amongst the authors. Rather, we focussed on assessing the concordance between scoring by conventional microscopy and by the digital images from the same sections. In the routine setting, inter-observer variation is a potential issue in IHC analyses. Digital imaging with automated scoring may be able to reduce the variability of scoring of IHC. Further research and development into such systems is urgently required.

## Conclusions

We have shown that digital microscopy images can be used as a high-throughput technique for assessing the immunohistochemical expression of proliferation markers in early invasive breast cancer with results that are comparable to those from light microscopy-based scoring. We used MCM2, Ki67 and Geminin, and found MCM2 to be the most sensitive marker of proliferation and prognosis among the three. Despite not finding these three markers to be independently prognostic of BCSS as evinced by our multivariable analysis, digital microscopy-based assessment of these and others may yet find utility in particular subgroups of breast cancer patients, for example in lymph node-negative, hormone receptor-positive patients, which have generally better prognoses. Future studies using immunohistochemistry should be directed towards utility of Ki67 and MCM2 in choosing the appropriate adjuvant therapy in early breast cancer cases.

## Additional files

Additional file 1: **Univariable breast cancer-specific analyses among 225 ER-positive cases of invasive breast cancer.** Kaplan Meier curves showing breast cancer-specific survival (BCSS) in relation to high (solid line) and low (dotted line) expression of Ki67 (A), MCM2 (B) and Geminin (C). The cut-offs of percentage expression were 8, 12 and 2.33 for Ki67, MCM2 and Geminin, respectively. Log rank *p*-values are stated. The number of patients at risk for every 2.5 years is given for each subgroup.
Additional file 2:
**Univariable and multivariable analyses of prognostic factors for 15-year breast cancer specific survival in 225 cases of ER+ breast cancer.**

Additional file 3:
**Sweave file containing reproducible report forestablishing the immunohistochemical score cut-offs that maximise prognostic accuracy.**
