# Supplementary material for: Digital imaging in the immunohistochemical evaluation of the proliferation markers Ki67, MCM2 and Geminin, in early breast cancer, and their putative prognostic value
Source: BMC Cancer. 2015 Jul 25;15:546. doi: 10.1186/s12885-015-1531-3 (PMC4513675; doi:10.1186/s12885-015-1531-3)
Supplement: Additional file 1: — Univariable breast cancer-specific analyses among 225 ER-positive cases of invasive breast cancer. Kaplan Meier curves showing breast cancer-specific survival (BCSS) in relation to high (solid line) and low (dotted line) expression of Ki67 (A), MCM2 (B) and Geminin (C). The cut-offs of percentage expression were 8, 12 and 2.33 for Ki67, MCM2 and Geminin, respectively. Log rank p-values are stated. The number of patients at risk for every 2.5 years is given for each subgroup. [file 12885_2015_1531_MOESM1_ESM.pdf]

Additional File 1

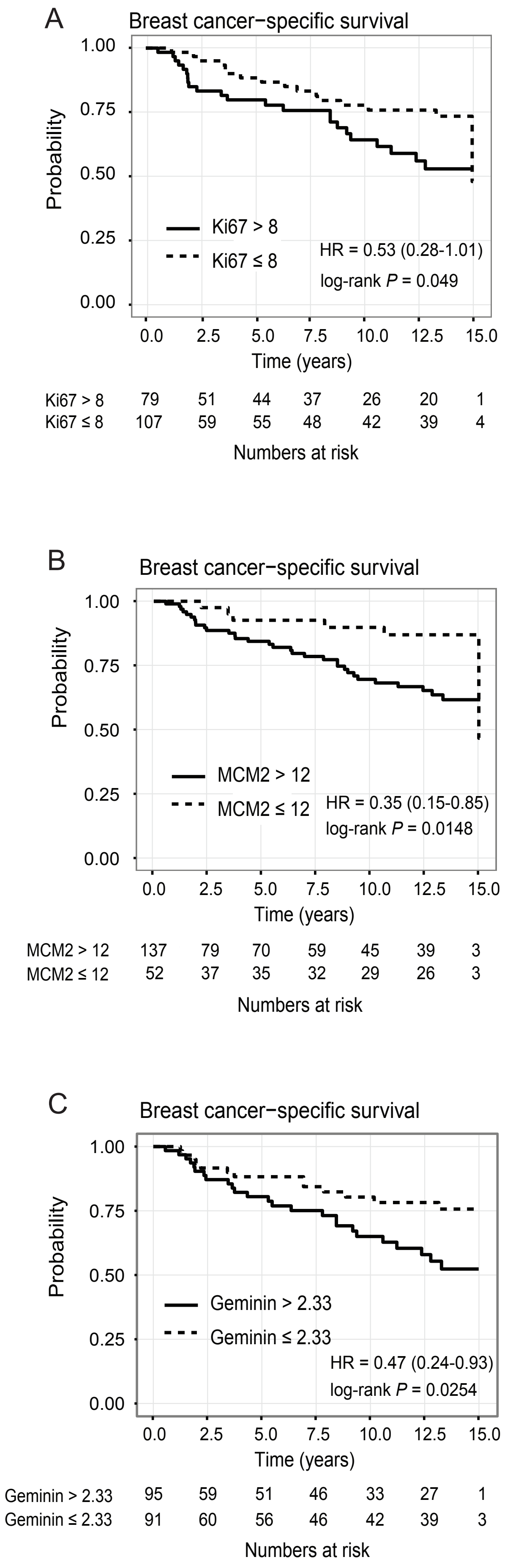

Additional File 1 Univariable breast cancer-specific analyses among 225 ER-positive cases of invasive breast cancer. Kaplan Meier curves showing breast cancer-specific survival (BCSS) in relation to high (solid line) and low (dotted line) expression of Ki67 (A), MCM2 (B) and Geminin (C). The cut-offs of percentage expression were 8, 12 and 2.33 for Ki67, MCM2 and Geminin, respectively. Log rank p-values are stated. The number of patients at risk for every 2.5 years is given for each subgroup.
