# Supplementary material for: Digital imaging in the immunohistochemical evaluation of the proliferation markers Ki67, MCM2 and Geminin, in early breast cancer, and their putative prognostic value
Source: BMC Cancer. 2015 Jul 25;15:546. doi: 10.1186/s12885-015-1531-3 (PMC4513675; doi:10.1186/s12885-015-1531-3)
Supplement: Additional file 2: — Univariable and multivariable analyses of prognostic factors for 15-year breast cancer specific survival in 225 cases of ER+ breast cancer. [file 12885_2015_1531_MOESM2_ESM.docx]

**Additional File 2: Univariable and multivariable analyses of prognostic factors for 15-year breast cancer specific survival in 225 cases of ER+ breast cancer**

| **Prognostic factor** | | **Univariable Cox regression analysis** | | | **Multivariable Cox regression analysis** | | |
| --- | --- | --- | --- | --- | --- | --- | --- |
|  | | **HR** | **95% CI** | **p-value** | **HR** | **95% CI** | **p-value** |
| **Ki67: low** | | 0.53 | 0.28-1.01 | 0.049 | 0.92 | 0.28-3.07 | 0.898 |
| **MCM2: low** | | 0.35 | 0.15-0.85 | 0.0148 | 0.49 | 0.07-3.52 | 0.477 |
| **Geminin: low** | | 0.47 | 0.24-0.93 | 0.0254 | 0.93 | 0.28-3.07 | 0.904 |
| **Histological grade: 3** | | 2.06 | 1.34-3.17 | 0.00348 | 1.22 | 0.43-3.47 | 0.71 |
| **HER2 status: positive** | | 1.95 | 0.45-8.34 | 0.361 | 0.53 | 0.06-4.73 | 0.57 |
| **LN status: positive** | | 4.52 | 2.1-9.73 | <0.0001 | 6.12 | 1.91-19.6 | 0.00227 |
| **Age > 50 yrs** | | 0.97 | 0.5-1.87 | 0.921 | 1.47 | 0.44-4.97 | 0.534 |
| **Tumour size: medium** | | 0.5 | 0.15-1.73 | 0.535 | 2.17 | 0.22-21.36 | 0.506 |
| **Tumour size: small** | | 0.53 | 0.16-1.76 | 0.535 | 2.40 | 0.22-26.2 | 0.472 |
| **After backward stepwise regression** | | | | |  |  |  |
| **Age > 50 yrs** | 2.26 | | 0.79-6.42 | 0.127 |  |  |  |
| **LN status: positive** | 7.13 | | 2.32-21.89 | 0.000594 |  |  |  |
