# Supplementary material for: Digital imaging in the immunohistochemical evaluation of the proliferation markers Ki67, MCM2 and Geminin, in early breast cancer, and their putative prognostic value
Source: BMC Cancer. 2015 Jul 25;15:546. doi: 10.1186/s12885-015-1531-3 (PMC4513675; doi:10.1186/s12885-015-1531-3)
Supplement: Additional file 3: — Sweave file containing reproducible report forestablishing the immunohistochemical score cut-offs that maximise prognostic accuracy. [file 12885_2015_1531_MOESM3_ESM.pdf]

Immunohistochemical evaluation of proliferation markers is consistent between digital and conventional imaging in breast cancer

—

Proliferation marker cut-off determination by maximising the balanced accuracy for the prediction of breast cancer-specific survival

—

Johnathan Watkins, March 2014

****

Proliferation status is believed to be important in the prognostication of early breast cancer. This is routinely determined through the assessment of mitotic count as a component of histological grade. Immunohistochemical assessment of markers of proliferation may provide additional prognostic information. We studied the immunohistochemical expression of three different markers of proliferation, Ki67, Mini-Chromosome Maintenance proteins (MCM2) and Geminin by conventional light microscopic and digital imaging. Levels of expression were then correlated with clinico-pathological features in a series of patients with long term follow-up. Our study cohort comprised tissue microarrays from 309 consecutive cases of invasive breast cancer assessed in triplicate. The expression of these markers was assessed on conventional light microscopic slides and on digital images; there was strong agreement between the two methods. Higher levels of all three markers of proliferation were significantly associated with high histological grade ($P < 0.001$) and ER-negativity ($P < 0.001$). MCM2 was the most sensitive marker of proliferation of the three, since it can be detected in cells during all phases of the cell cycle and is seen at higher frequency. In contrast, the low frequency of expression of geminin make it the least sensitive marker for assessment of differences in the proportion of proliferating cells between lesions. By establishing optimal cut-offs to group tumours into high or low expressing for each, all three proliferation markers were predictive of 15-year breast cancer specific survival (BCSS) in univariate Cox regression analyses. However, in multivariate analysis none of the markers were significantly associated with BCSS; whilst lymph node status (HR = 3.9, 95% CI = 1.79-8.5, P = 0.0006) and histological grade (HR = 1.84, 95% CI = 1-3.38, P = 0.05) remained significantly predictive. In this study, we reveal the dissimilarities in the frequencies, and also the value with respect to survival prediction, of these different proliferation markers. We demonstrate high concordance between the levels of expression of the proliferation markers assessed using conventional light microscope and digital imaging.

****

1. PACKAGE INSTALLATION

```
>   #install.packages("ggplot2")
>   #install.packages("ROCR")
>   #install.packages("verification")
>   #install.packages("survivalROC")
```

****

2. FUNCTION READ-IN
The following function calculates the cut-off that maximises the balanced accuracy. Second, it plots ROC curves followed by plots of cut-off vs balanced accuracy. The recommended cut-off is shown in these second plots:

```
> calculateROCBalAccCutoff <- function(score, dataset, output.f, predict.time,
+                                       time="t.rfs", event="e.rfs") {
+
+   library("ggplot2")
+   library("ROCR")
+   library("verification")
+   library("survivalROC")
+
+   # pred <- with(d,prediction(MCM_avg,e.pfs))
+   # perf <- performance(pred,"tpr", "fpr")
+   # auc <-performance(pred, measure = "auc")@y.values[[1]]
+
+   surv.pred <- survivalROC(dataset[,time], dataset[,event], dataset[,score],
+                            predict.time=predict.time, method='KM')
+   rd <- data.frame( x=surv.pred$FP,y=surv.pred$TP )
+   bal.accuracy <- (surv.pred$TP + (1-surv.pred$FP))/2
+
+   p <- ggplot(rd,aes(x=x,y=y)) + geom_path(size=0.9)
+   p <- p + geom_segment(aes(x=0,y=0,xend=1,yend=1),colour="black",linetype= 2)
+   p <- p + geom_text(aes( x=1,
+                           y= 0.05, hjust=1, vjust=0,
+                           label=paste(sep = "", "AUC = ", round(AUC,3) )),
+                      data=as.data.frame(surv.pred), colour="black", size=8)
+   p <- p + scale_x_continuous(name= "1 - Specificity")
+   p <- p + scale_y_continuous(name= "Sensitivity") # TPR
+   p <- p + opts(
+     axis.text.x = theme_text(size = 20,colour="black"),
+     axis.text.y = theme_text(size = 20,colour="black"),
+     axis.title.x = theme_text(size = 25,colour="black"),
+     axis.title.y = theme_text(size = 25,colour="black"),
+     legend.position = "none",
+     legend.title = theme_blank(),
+     panel.background = theme_blank(),
+     panel.grid.minor = theme_blank(),
+     panel.grid.major = theme_line(colour='lightblue'),
+     plot.background = theme_blank()
+   )
+
+   #pdf(paste(output.f, score, "_", predict.time, ".", event, ".pdf", sep=""),
+   #    width=12, height=10)
+   par(las=1, mar=c(6,8,4,4))  #mfrow=c(2,1),
+
```

```
+   #print(p)
+
+   p1 <- plot(surv.pred$cut.values, bal.accuracy, type="b", pch=17, lwd=2,
+        xlab=paste(score,"score"), ylab="Balanced Accuracy",cex.lab=2,
+        cex.axis=1.75, mgp=c(4.5,1,0))
+   optim.cutoff <- round(surv.pred$cut.values[which.max(bal.accuracy)],3)
+   p1 <- abline(v=optim.cutoff, lwd=2.5, lty=2, col="darkred")
+   p1 <- mtext( paste("Optimal cutoff = ", optim.cutoff, sep=""), 3,line=1, outer=F,
+          cex=2, col="darkred")
+   print(p1)
+   #dev.off()
+   optim.cutoff
+
+ }
```

****

3. CALCULATION OF CUT-OFFS
The table 'data.IHC.txt', which is read in, contains clinicopathological, immunohistochemical and breast cancer-specific survival data:

```
> data.IHC <-read.delim("~/Desktop/ProliferationBiomarking/MGKB/results/tables/data.IHC.txt")
> data.IHC.bcss <- data.IHC[ which(!is.na(data.IHC$t.bcss) &
+                                     !is.na(data.IHC$e.bcss) ), ]
> scores <-c("KI_avg", "MCM_avg", "GEM_avg")
```

Next, the cut-off that maximises the balanced accuracy is calculated along with plotting of ROC curves and balanced accuracy plots:

```
> optim.cutoffs.bcss <- matrix(NA, length(scores), 1,
+                               dimnames=list( c(scores), c("15yr survival") ) )
> for (score in scores) {
+
+   optim.cutoffs.bcss[ score, "15yr survival"] <-
+       calculateROCBalAccCutoff(score=score, dataset=data.IHC.bcss,
+                                output.f="results/plots/roc.curves/",
+                                predict.time=15, time="t.bcss", event="e.bcss")
+
+ }

 51 records with missing values dropped.
NULL

 49 records with missing values dropped.
NULL

 51 records with missing values dropped.
NULL
```

```
> print(optim.cutoffs.bcss)

        15yr survival
KI_avg          8.000
MCM_avg        12.000
GEM_avg         2.333
```

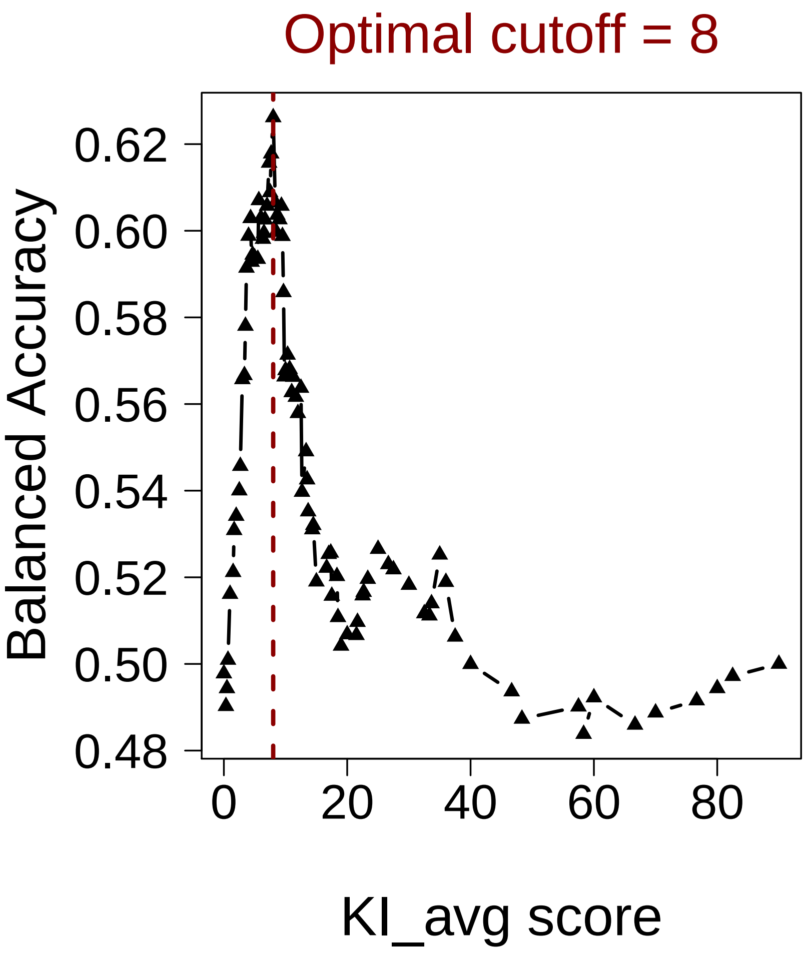

Figure 1. Representative cut-off vs balanced accuracy plot for the Ki67 marker
